# Investigating the phenotypic alterations associated with hypermucoviscous hypervirulent *Klebsiella pneumoniae* during phage resistance development

**DOI:** 10.1186/s12866-025-04268-x

**Published:** 2025-08-22

**Authors:** Ramya Juliet, Ramesh Nachimuthu

**Affiliations:** Antibiotic Resistance and Phage Therapy Laboratory, Centre for Advanced Research in Bacteriophage and Infectious Diseases, School of Bio Sciences and Technology, Vellore Institute of Technology (VIT), Vellore, 632014 Tamil Nadu India

**Keywords:** Antibiotic resistance, Phage resistance, *Klebsiella pneumoniae*, Hypermucoviscous, Phage Therapy, Biofilms

## Abstract

Phage therapy has been explored and used compassionately in the post-antibiotic era, though phage resistance might pose a serious challenge. The advent of hypervirulent and hypermucoviscous traits in *Klebsiella pneumoniae* limits therapeutic choices. This study investigated the phage resistance in hypermucoviscous hypervirulent *Klebsiella pneumoniae* clinical strain Kleb_53. A Klebsiella phage Disc against the Kleb_53 strain was isolated from sewage. The phage exhibited stability between − 20 °C and 60 °C and within the pH range of 3 to 11. The phage adsorption time was 15 min, with a latent period of 30 min and a burst size of 354 virions. The phage-resistant Kleb_53 variants were screened and examined for their phenotypic variations, antibiotic susceptibility, and biofilm formation. Colony morphotype variants were observed, including smooth, rough, and small colony variants. String, aggregation, and wetness tests confirmed reduced mucoviscosity. The plaque morphology differed between the wild and variants. Additionally, resistance to meropenem and third-generation cephalosporins was reversed, whereas the biofilm-forming ability varied among the recovered variants. This study demonstrates that ongoing phage-host interactions drive phenotypic changes and the emergence of phage-resistant variants with altered antibiotic susceptibility and biofilm-forming capacity. It also underscores the need for further research on phage resistance and strategies to overcome it for the effective application of phage therapy.

## Introduction

Bacteriophages (phages) and bacteria have existed together and interdependently evolved for eons, shaping each other’s evolutionary trajectories through complex interactions [1]. Phages are prokaryotic viruses that infect and kill bacteria by binding to the cell wall receptor, such as the proteins, polysaccharides, capsules, pili, or flagella, of the host bacteria through their tail spikes or tail fiber proteins [2, 3]. Following the initial reversible binding and irreversible attachment, the phage ejects its genome into the host [3], initiating either the lytic cycle, resulting in bacterial killing, or a lysogenic cycle where the phage genome integrates into the host as a prophage or plasmid [1].

Bacteria have evolved various adaptations to counter each stage of phage infection. One of the primary defense mechanisms is phenotypic variation, which can significantly hinder phage adsorption. Bacteria prevent phage adsorption by modifying/losing the phage receptor proteins on their cell surface, forming biofilms, and producing capsules or decoy receptors. Other mechanisms include abortive infection (phage-infected bacteria self-destruct to protect their bacterial population), restriction-modification system, superinfection exclusion, and CRISPR-Cas [4]. The ongoing co-evolution between phages and bacteria can follow two major phases. Either it occurs through an arms race, marked by repeated cycles of resistance and sensitivity, or through directional evolution, where ongoing mutations in both organisms lead to phages with expanded host ranges and bacteria capable of resisting multiple phages [5]. Changes in gene expression regulation are often transient and driven by selective pressure; this reversible phenomenon is known as phase variation [3].

Adaptations associated with phage resistance often lead to phenotypic changes, such as the loss of lipopolysaccharides, capsule, mucosity, and alterations in biofilm formation. These changes can restore antibiotic sensitivity and increase bacterial vulnerability to the host immune response [6]. This phenomenon, often referred to as a fitness trade-off, highlights how resistance to phages may come at the cost of susceptibility to antibiotics and host immunity [7]. Antibiotic resistance and phage resistance are bacterial defense mechanisms that help them survive in challenging ecological pressures [8]. Although phage therapy has been gaining interest, the area of phage resistance in bacteria, particularly the associated phenotypic and genotypic adaptations, remains largely unexplored.

*Klebsiella pneumoniae* is a major gram-negative pathogen and the second leading cause of nosocomial infections worldwide [9]. It exhibits two major pathotypes: classical (cKp) and hypervirulent (HvKp). The classical form is typically linked to multidrug resistance and hospital-acquired infections, while the hypervirulent form is characterized by enhanced virulence and is more commonly associated with community-acquired infections [10]. The emergence of hypermucoviscous hypervirulent *K. pneumoniae* (HmHvKp) poses a growing threat due to its enhanced capsule production, iron acquisition capabilities, and increasing antibiotic resistance, particularly to carbapenems, resulting in high mortality rates ranging from 11.9 to 37.1% [11–13].

Given the rise of drug-resistant HmHvKp, Klebsiella-specific phages have gained attention as potential therapeutic agents [14, 15]. These phages often encode depolymerases capable of degrading the overproduced capsule and exopolysaccharides, allowing them to effectively infect hypervirulent strains that are otherwise difficult to treat with antibiotics [16]. This study investigates the phenotypic alterations that occur in the hypermucoviscous hypervirulent *K. pneumoniae* (HmHvKp) during the development of phage resistance, especially focusing on changes in colony morphology, capsule production, and virulence traits that may impact phage therapy effectiveness.

## Methodology

### Bacterial identification and characterization

A clinical strain of *K. pneumoniae*, Kleb_53, was collected from a diagnostic center in Chennai, Tamil Nadu, India. Initially, the strain was identified using the VITEK identification system and later confirmed by 16 S rRNA sequencing. The universal primers 27 F (5′-AGAGTTTGATCCTGGCTCAG-3′) and 1492R (5′-GGTTACCTTGTTACGACTT-3′) were used, and the amplified product was sequenced and deposited in GenBank (Accession no PV029729) [17]. The hypermucoviscosity of the clinical strain was tested using the string test [18]. Biofilm formation of the Kleb_53 clinical strain was quantified using the CV assay and categorized using the protocol described previously [19].

The micro broth dilution method was performed for the three last resort antibiotics, meropenem, colistin, and tigecycline, following Clinical and Laboratory Standards Institute (CLSI, 2023) to determine the minimum inhibitory concentration [20]. The resistance genes *bla*_NDM_, *bla*_OXA−48 like_, and *bla*_KPC_ in Kleb_53 were screened using the primers F- GCAGCTTGTCGGCCATGCGGGC, R- GGTCGCGAAGCTGAGCACCGCAT; F- GCGTGGTTAAGGATGAACAC, R-CATCAAGTTCAACCCAACCG; F- TGTCACTGTATCGCCGTC, R- CTCAGTGCTCTACAGAAAACC, respectively [21] and the virulence genes were screened using the primers aerobactin F- 5′-GCATAGGCGGATACGAACAT-3′ and R- 5′-CACAGGGCAATTGCTTACCT-3′, *rmpA* gene F-5′-ACTGGGCTACCTCTGCTTCA-3′ and R- 5′-CTTGCATGAGCCATCTTTCA-3′ [22, 23]. All the PCR conditions were described previously [21, 22].

### Bacteriophage isolation

Using the phage enrichment technique, Kleb_53 was used as a host to isolate bacteriophage from sewage water collected from Vellore, Tamil Nadu, India. Briefly, sewage was added to a logarithmic phase bacterial culture and incubated overnight at 37° C, 120 rpm. After incubation, the mixture was centrifuged at 7000 rpm for 15 min [24]. The supernatant was filtered by a 0.22-µm pore-sized membrane syringe filter and tested for the presence of phage through a spot test. Briefly, 10 µl of supernatant from enrichment was spotted on a bacterial lawn of Kleb_53 in Luria Bertani (LB) agar medium (Hi Media, India) and incubated at 37 °C. The presence of bacterial clearance indicates a positive spot test. Double agar overlay (DAOL) was performed to confirm the presence of phages and observe plaque morphology. In DAOL, 200 µl of the host, 100 µl of supernatant, and 4 ml of molten soft agar (0.45%) were mixed, poured onto the hard agar plate (2%), and incubated at 37 °C until the appearance of plaques [24]. The clear, transparent plaques represent the phages’ lytic nature, whereas the turbid plaques represent their lysogenic nature.

### Purification and multiplication of the phage

A clear lytic plaque was selected from the DAOL plate, and purified using the pickate method to obtain a homogenous population of phages. 4 mL of SM buffer [5.8 g NaCl, 50 mL 1 M Tris–HCl (pH 7.5), 2 g MgSO_4_.7H_2_O, and 5 mL of 2% gelatin for 1,000 mL] was flooded onto the plate containing uniform plaque morphology, and incubated for 4 h at 4 °C. The buffer was collected from the plate and centrifuged at 7000 rpm for 10 min. The supernatant was added to the logarithmic phase bacterial culture, allowed to multiply for 24 h, centrifuged, and filtered using a 0.22 μm membrane syringe filter [17]. The phage titer was calculated in plaque-forming units per milliliter (PFU/mL) using the agar overlay method and stored at 4 °C until further use.

### Host range and stability

The host range of the phage was identified by performing a spot test against 145 non-repetitive clinical isolates of *Klebsiella* species. The phage stability at various temperatures and pH was determined. Briefly, 100 µL of the phage lysate (10^10^ PFU/mL) was incubated at different temperatures such as − 20°, 4°, 20°, 30°, 37°, 50°, 60°, and 70 °C and with various pH 3, 5, 7, 9, and 11 for 1 h and the titer was determined using DAOL. The experiment was performed in triplicate, and the graph was plotted [24].

### Microscopic examination

Transmission electron microscopy (TEM) was used to examine the phage morphology. Briefly, 10 µl of phage (10^10^ PFU/mL) was coated onto a copper grid. The grid was negatively stained using 1% (w/v) uranyl acetate, washed twice with sterile water to remove excess stain, and observed under a transmission electron microscope (FEI-TECNAI G2-20 TWIN, VIT, Vellore) [24].

### Phage adsorption and one-step growth curve

The phage's time to adsorb to the bacterial cell is called adsorption. Briefly, to a logarithmic phase bacterium (OD-0.08), 10^6^ PFU/mL phage lysate (Multiplicity of Infection, MOI= 0.01) was added, aliquots were taken at 3 minute time intervals for 15 min, treated with 1 % chloroform to eliminate the bacteria, and DAOL was performed to quantify the number of free phages. In the one-step growth curve, the latency period (time taken by the adsorbed phage to lyse the host) and burst size (number of phages released per bacterial cell) were calculated. Briefly, the phage (MOI 0.01) and host mixture were incubated for 15 min (pre-determined adsorption time) at 37°C, centrifuged at 10,000 rpm for 5 min, the supernatant containing the free phages was discarded, and the pellet was resuspended in broth. DAOL was performed every 10 min up to 1 hour to titrate the number of adsorbed phages. Burst size is the number of phage particles released per infected bacterial cell, calculated by subtracting the initial PFU/mL from the first burst [24]. All experiments were conducted in triplicate, and data were analyzed and plotted using GraphPad Prism software.

### Acquisition of phage-resistant variants

The phage-resistant variants were obtained using the modified protocol [25]. Briefly, 10^10^PFU/mL phage was added to the host bacterium Kleb_53 (wild-type, 10^8^ CFU/mL) and incubated overnight at 37 °C, 120 rpm. Centrifuged at 6500 rpm for 10 min, the supernatant (containing the phage) was removed, and the pellet (bacteria) was washed twice with SM buffer and resuspended in the LB broth. 100 µl of resuspended bacteria were spread plated on LB and Mac Conkey agar plates using sterile L-rod, the plates were incubated at 37 °C, and observed for colony morphology variations. The 10^10^ PFU/mL fresh phage was again added to the resuspended pellet containing the phage-interacting host and incubated overnight, centrifuged, and a spread plate was performed, and the protocol was repeated for 5 days. Three similar colonies of each morphotype were stabbed and stored. For every experiment, the culture was recovered from the stab to avoid repeated subculturing and loss of acquired characteristic phenotype.

### Variant growth dynamics

The growth pattern of the Kleb_53 morphotype variants obtained from wild type and phage interaction was determined by measuring optical density readings. The overnight culture was diluted to 0.1 OD and incubated at 37 °C, 120 rpm. Optical density readings were taken at 2 h intervals for 24 h at a wavelength of 600 nm with a spectrophotometer (UV-1280, Shimadzu) [26]. The experiments were performed in triplicate for each morphotype, and the graph was plotted based on the mean ± SD values.

### Phenotypic methods for assessing the morphological variations in phage-resistant variants

#### Plaque morphology variations

All the sub-cultured colonies from the spread plate were inoculated in LB broth, incubated, and adjusted to a 0.5 McFarland turbidity standard. A spot test was performed against each colony variant. The colonies were categorized into two, based on their phage sensitivity. The presence of lysis in the spot test, regardless of observed colony morphology changes, was classified as a phage-sensitive variant (PSV). Variants that did not exhibit lysis in the spot assay were defined as phage-insensitive/resistant variants (PRV). Thus, morphological variation alone was not used to infer phage resistance, as some variants with altered appearance remained susceptible to phage-mediated lysis. Since this study involves only phenotypic observation, the colonies were named as variants, not mutants. The phage-sensitive variants were subjected to double agar overlay to observe for plaque morphology variations. The efficiency of plaquing (EOP) was also examined for the phage-sensitive variants using a double agar overlay.

#### String test

All the colony variants were examined for the hypermucoviscous phenotype. Briefly, a fresh overnight-grown colony was gently touched with the loop and extended to observe the formation of the viscous string. If the string formed is above 5 mm, it’s considered to be positive for the string test [18].

#### Aggregation index

A single colony was inoculated into LB broth from each morphotype variant and incubated overnight at 37°C, 120 rpm. After incubation, the early stationary phase cultures were subjected to slow-speed centrifugation at 2500 rpm for 5 min. The supernatant was transferred to a new tube. The pellet/ sediment was resuspended in broth. The optical density readings of the supernatant and resuspended pellet were measured at 600 nm. The aggregation index was calculated based on the given formula [27]. The experiment was performed in triplicate for each of the three colonies belonging to the same colony morphotype, and the graph was plotted based on the mean value. 

 •Aggregation index= Supernatant OD/ Sediment OD

#### Wetness assay

A wetness assay was performed for all the variants to determine the wetness of the colony in a swarming plate (0.4% agar). All the variants and wild-type were spotted at a concentration of 10^8^ CFU/mL and incubated overnight. A capillary tube was placed on the fresh-grown colony for 10 s. The amount of wetness drawn in the capillary tube is measured in millimeters [28]. The experiment was performed in triplicate for each morphotype.

#### Antibiotic susceptibility testing

Kirby Bauer disk diffusion assay was performed for the variants to observe any changes in their susceptibility pattern compared to the wild type. The following antibiotics were used for screening: meropenem (MRP), imipenem (IPM), cefotaxime/clavulanic acid (CEC), ceftriaxone (CTR), ceftazidime (CAZ), piperacillin-tazobactam (PIT), ciprofloxacin (CIP), and gentamicin (GEN). Briefly, a lawn of culture adjusted to 0.5 McFarland was swabbed on a Mueller Hinton agar plate, disks were placed, and incubated for 16–18 h at 37 °C [29]. The zone of inhibition was recorded, the experiments were replicated independently, and the mean values were interpreted based on CLSI guidelines [20].

#### Biofilm formation

Biofilm formation of all the colony morphotype variants was quantified using a crystal violet (CV) assay. Briefly, 100 µl of 10: 100 diluted overnight culture was added to the 96-well flat-bottomed microtiter plate and incubated for 24 h at 37 °C. Post-incubation, the planktonic cells were removed, washed twice, and stained with 0.1% crystal violet for 15 min. The stain was removed, and the wells were washed twice. The CV stain was dissolved using 33.3% glacial acetic acid. The optical density reading was taken at 570 nm (BioTek India) [30]. Wild-type biofilm served as a positive control, and only broth as a negative control. The experiments were conducted in triplicate, and the graph was plotted.

### Statistical analysis

All the experiments were performed in triplicate, and the data were represented as the mean ± standard deviation (SD). The results of the stability, aggregation index, wetness assay, and biofilm formation experiments were analyzed using a t-test and one-way ANOVA. The results were considered significant if p < 0.05. All the data analysis was carried out in GraphPad Prism 8.0 software.

## Results

### Characteristics of the host strain kleb_53

The clinical strain Kleb_53 used in this study tested positive for the string test (string formed > 5 mm) and was found to be a moderate biofilm producer (OD-0.76; ODC-0.25) based on the criteria established by Loganathan et al., 2022, where moderate producers are defined as 2×ODC < OD ≤ 4×ODC. The strain resisted all three antibiotics tested in MIC: meropenem, colistin, and tigecycline (all ≥ 4 µg/mL). There was no presence of the screened resistance genes. Aerobactin (GenBank accession number PP994840) and rmpA (GenBank accession number PP994850) genes were present. Therefore, the strain Kleb_53 was classified as hypervirulent (Hv) and hypermucoviscous (Hm) Klebsiella pneumoniae (HvHmKp).

### Isolation and morphological analysis

**Fig. 1:**
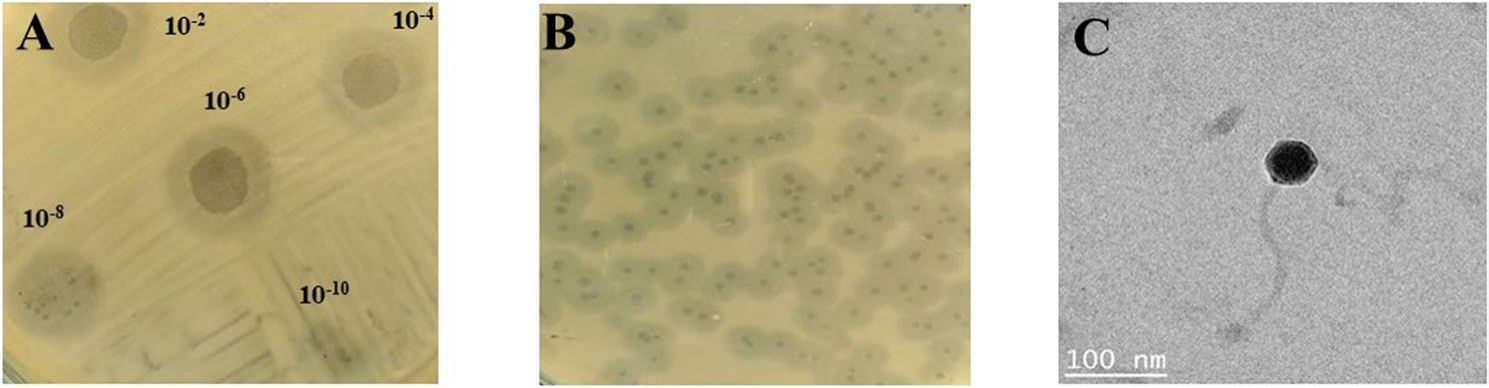
Morphological characterization of the Disc phage. **A** Spot test plate showing dilution spots of the Disc phage lysate. **B** Double agar overlay plate displaying clear, circular lytic plaques with 4 mm semi-translucent halo zones, indicating depolymerase activity. **C** Transmission electron microscopy (TEM) image of the Disc phage revealing T5-like morphology, characterized by an icosahedral head and a long non-contractile tail

A lytic bacteriophage, named Klebsiella phage Disc (hereafter referred to as ‘Disc’), was isolated from a sewage water sample using the Kleb_53 strain as the host. The phage was subsequently purified and propagated using the same host strain throughout the study. The plaque was clear, circular, and transparent with a halo zone of 4 mm, indicating depolymerase activity. TEM analysis of the Disc phage showed a T5-like morphology, with a 50 ± 3.0 nm icosahedral head and 170 ± 3.0 nm long non-contractile tail (Fig. 1C).

### Host range and stability

**Fig. 2:**
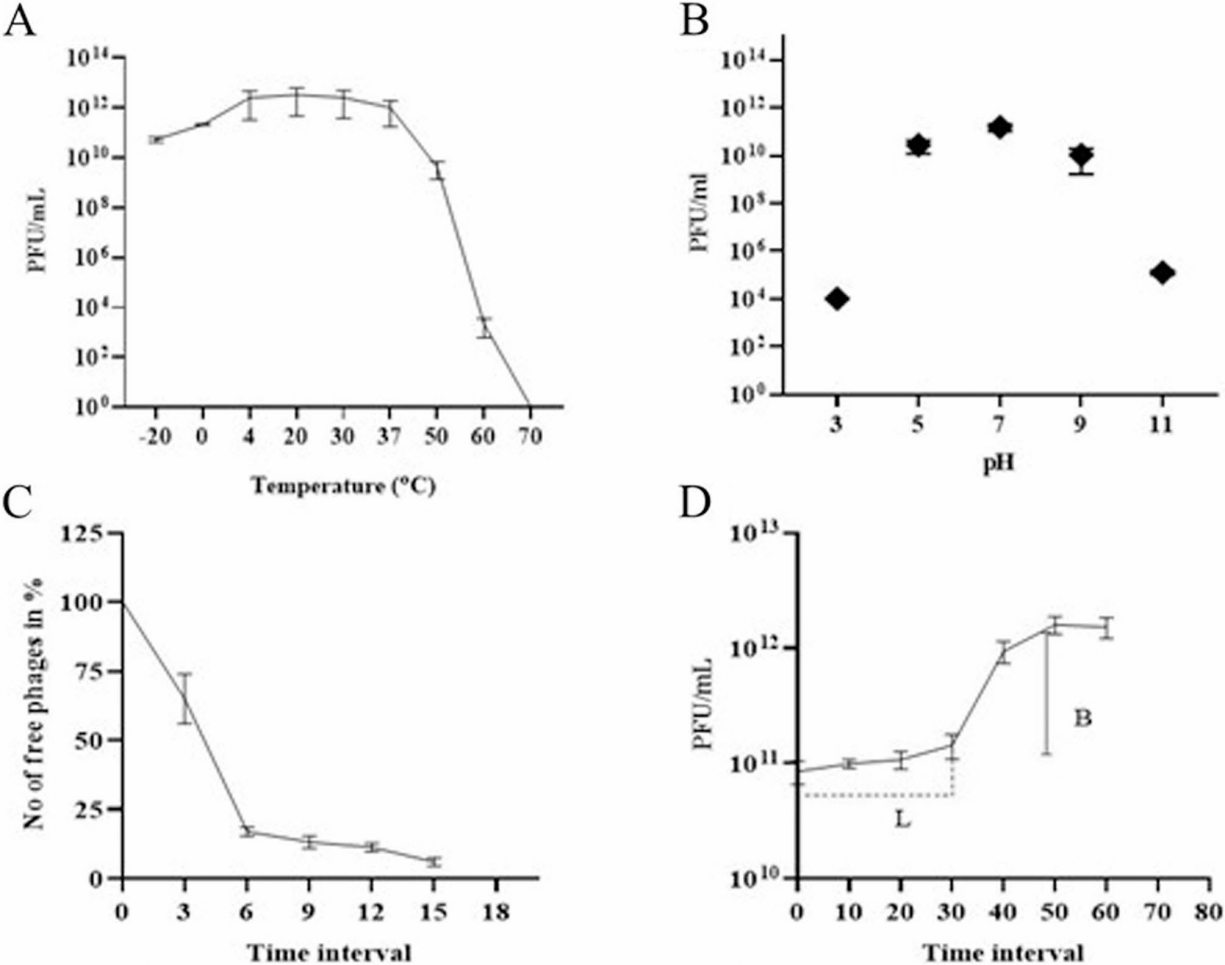
The biological characteristics of the Disc phage. **A** Thermal stability, showing no significant difference in titer between − 20 °C and 37 °C (*p* > 0.05), with reductions observed at 50 °C (*p* = 0.02), 60 °C (*p* = 0.0002), and complete inactivation at 70 °C (*p* < 0.0001). **B** pH stability, showing high stability at pH 7 and moderate stability at pH 5 (*p* = 0.0002) and pH 9 (*p* = 0.01), with significant reductions at pH 3 and pH 11 (both *p* < 0.0001). **C** Phage life cycle focusing on adsorption, and (**D**) one-step growth curve showing a latency period (L) of 30 min and a burst size (**B**) of 354 phage virions per infected cell. All experiments were conducted in triplicate, and the data shown are represented as mean ± SD

The Disc phage was able to lyse and form plaques in 48 out of 145 *Klebsiella* clinical strains. The Disc phage remained stable between − 20 and 37 ˚C with no significant difference in the titer (p > 0.05). In contrast, it decreased at 50˚C (p = 0.02) and 60˚C (p = 0.0002) and was inactivated at 70˚C (p < 0.0001) (Fig. 2A). The Disc phage showed high stability at pH 7 and remained stable at pH 5 (p = 0.0002) and 9 (p = 0.01). In contrast, the titer was reduced at extreme acidic and alkaline conditions of pH 3 (p < 0.0001) and pH 11 (p < 0.0001) (Fig. 2B).

### Life cycle of disc phage

The Disc phage attained 84% adsorption to its host within 6 min and 94% adsorption in 15 min (Fig. 2C). In a one-step growth curve, the latent period of the phage was found to be 30 min with a rise period of 20 min. The Disc phage had a high burst size of 354 phages per infected cell (Fig. 2D).

### Phage-resistant variants

**Fig. 3:**
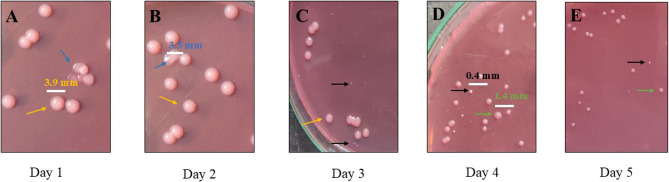
Spread plate representation of colony morphotype variants during phage resistance screening. **A**–**E** Spread plates showing the emergence of different colony morphotypes from Day 1 to Day 5 during the screening of phage-resistant variants. The colony diameters ranged from approximately 0.1–4 mm after 24 h of incubation on MacConkey agar. Arrow colors indicate morphotypes: orange – smooth (S), blue – rough (R), black – small colony variant-smooth (SCV-S), green – small colony variant-rough (SCV-R)

**Fig. 4:**
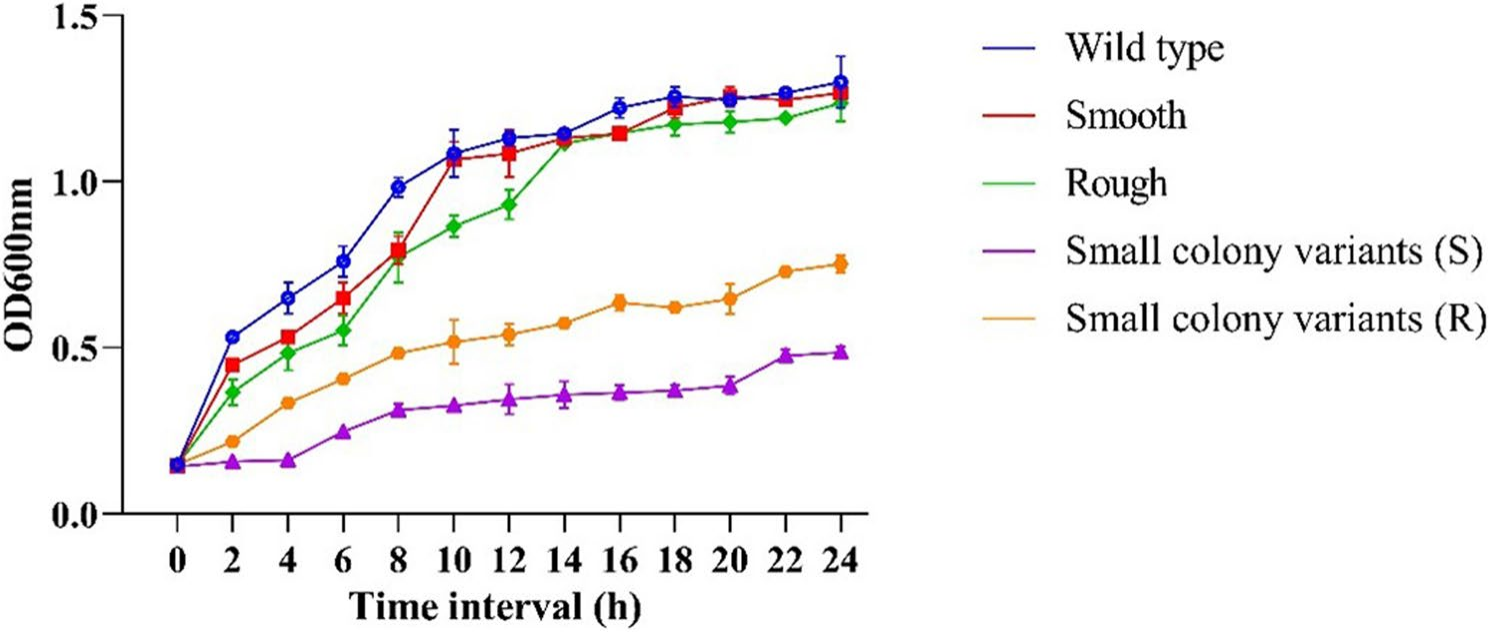
Bacterial growth curves of colony morphotype variants emerging during bacteria-phage interactions. Growth was monitored over time, and all experiments were performed in triplicate. Data are presented as mean ± standard deviation (SD)

Phages were allowed to interact with the same host inoculum for five consecutive days. The spread plate technique after each day revealed the variation in the host (wild type) colony morphology. Various colony morphotypes were observed during the study, such as rough dry irregular colony (R), smooth circular mucoid colony (S), and small colony variants (SCV-S, smooth and SCV-R, rough) (Fig. 3; Table 1). The bacterial growth dynamics of the variants showed deviation in the growth pattern from the wild type, wherein S and R colonies exhibited almost similar growth patterns. However, small colony variants couldn’t reach more than 0.8 OD, indicating lower cell densities, in which SCV-R had better growth than SCV-S (Fig. 4). After day 5, the colonies formed on the spread plate became unculturable, yet remained viable. This suggests a potential bacterial survival trade-off, although delayed growth due to phage-induced physiological burden or persistence mechanisms cannot be ruled out.

**Table 1:** Phenotypic variations observed in the wild-type colony during the screening of phage-resistant variants from day 1 to day 5. ✓ indicates the presence of a specific morphotype. Phage activity refers to the ability of the disc phage to lyse each variant in the spot test: ‘+’ indicates Lysis (phage-sensitive), and ‘–’ indicates no Lysis (phage-resistant/insensitive). The colony morphotypes are defined as follows: S – smooth, R – rough, SCV-S – small colony variant-smooth, and SCV-R – small colony variant-rough

Colony morphotype variants	Day 1		Day 2		Day 3		Day 4		Day 5	
	Present	Phage activity	Present	Phage activity	Present	Phage activity	Present	Phage activity	Present	Phage activity
S	⎫	+	⎫	+	⎫	+				
R	⎫	+	⎫	+						
SCV-S	⎫	-	⎫	-	⎫	-	⎫	-	⎫	-
SCV-R							⎫	+	⎫	+

### Plaque morphology variation

**Fig. 5:**
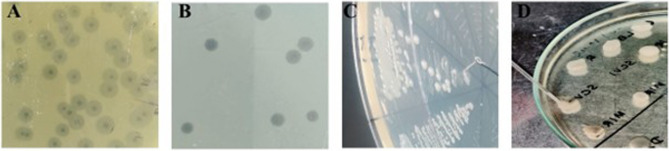
Properties of the different morphotypes of Kleb_53. **A** Double agar overlay using the wild-type Kleb_53 as host, showing distinct depolymerase activity. **B** Double agar overlay assay with the rough morphotype (R) as host, showing absence of depolymerase activity and a larger lytic zone. **C** Positive string test result for the wild-type *Kleb_53*, indicating hypermucoviscosity. **D** Capillary tube-based wetness assay assessing the mucosity of the colonies

Spot tests against the wild and variants revealed that smooth (S), rough (R), and rough small colony variants (SCV-R) were susceptible to the Disc phage and, therefore, regarded as phage-sensitive. Small colony variants (SCV-S) were non-susceptible and, therefore, considered phage-resistant/insensitive variants (Table 1). The efficiency of the plaquing analysis revealed that the Disc phage had low EOP (≤ 10^6^ PFU/mL) against the phage-sensitive variants compared to the wild type (1012 PFU/mL). DAOL showed differences in the plaque morphology. In Fig. 5B, the rough (R) morphotype has likely lost its capsule to develop resistance to the phage. This loss explains the absence of halo zones around the plaques, as there is no capsule available for degradation. Whereas with the wild type, the phage produced small lytic zone plaques with a distinct halo zone (Fig. 5A).

### Hypermucoviscous tests

**Fig. 6:**
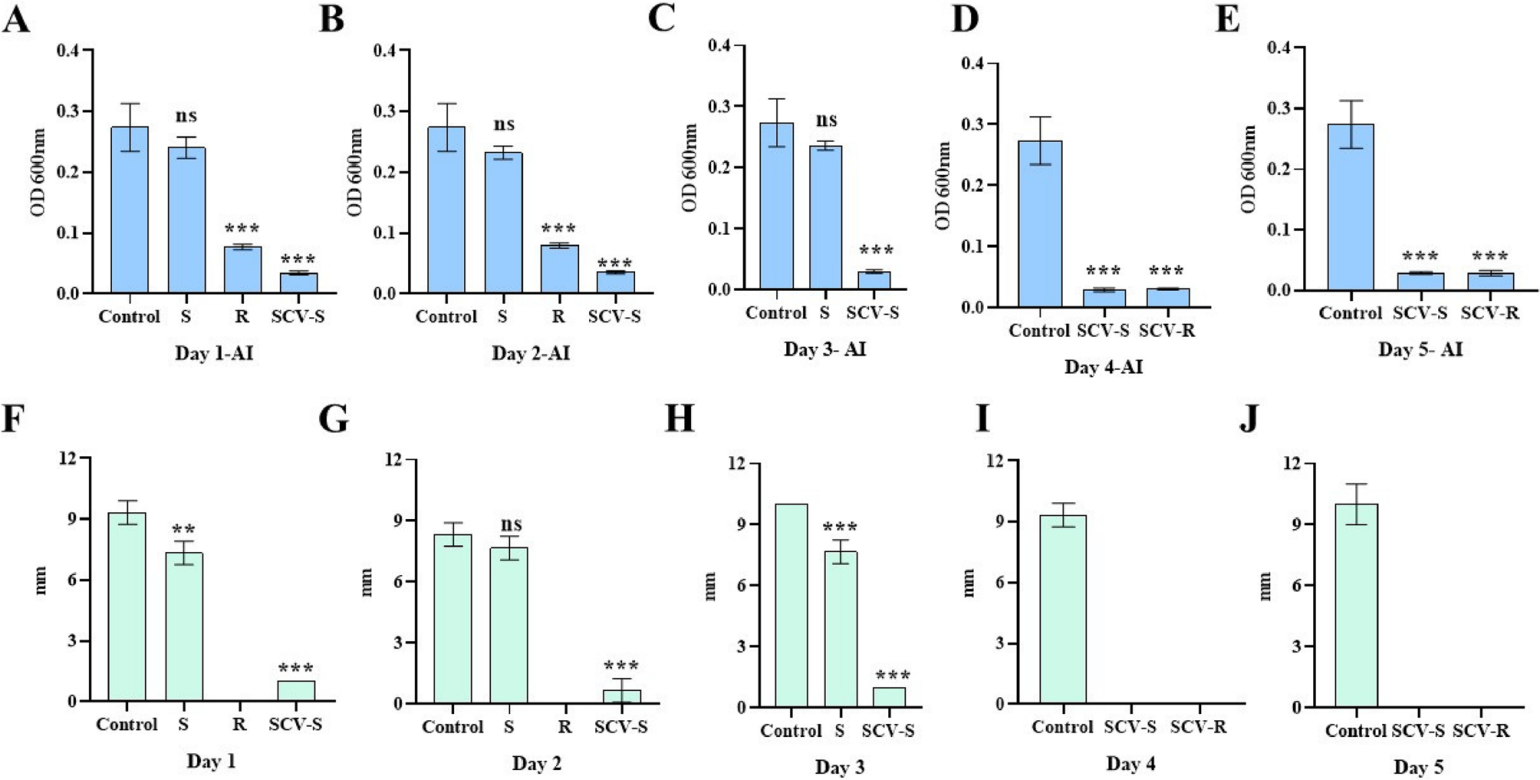
Assessment of phenotypic properties in colony morphotype variants (**A**–**E**) Aggregation index of the wild-type and colony morphotype variants from day 1 to day 5. (**F**–**J**) Wetness assay measurements (in mm) of the wild-type and colony morphotypes from day 1 to day 5. Control – wild-type Kleb_53; S – smooth; R – rough; SCV-S – small colony variant-smooth; SCV-R – small colony variant-rough. All experiments were performed in triplicate, and data are presented as mean ± standard deviation (SD). *p* ≤ 0.05 compared to the control. (ns = not significant, *p* ≤ 0.01, *p ≤ 0.001)

Only smooth colony variants (S) displayed positive results for the string test, similar to wild type (Fig. 5C), while rough and small colony variants tested negative. The aggregation index was high in the wild, followed by smooth, rough, and small colony variants (smooth and rough) (Fig. 6A and E). Wetness assay demonstrated that mucosity in the colonies has reduced significantly in the variants compared to the wild/control group (Fig. 5D, 6 F–6 J).

### Antibiotic susceptibility testing

The host strain Kleb_53 was resistant to the following antibiotics in the Kirby-Bauer disk diffusion test: meropenem, imipenem, cefotaxime/clavulanic acid, ceftriaxone, ceftazidime, piperacillin-tazobactam, ciprofloxacin, and gentamicin. The variants were also examined likewise and they displayed alterations in their susceptibility pattern. By the end of day 5, both smooth and rough small colony variants became sensitive to meropenem, ceftazidime, cefotaxime/clavulanic acid, ceftriaxone, and piperacillin/tazobactam. The results were tabulated and analyzed (Table 2).

**Table 2:** Representation of the antibiotic susceptibility pattern of all the colony morphotype variants obtained from day 1 to day 5, assessed using the Kirby-Bauer disk diffusion method. SCV- small colony variants

		MRP 10mcg	IPM 10mcg	CAZ 30mcg	CEC 30/10mcg	CTR 30mcg	GEN 10mcg	PIT 100/10mcg	CIP 30mcg
		≥ 23 20–22 ≤19 mm	≥ 23 20–22 ≤19	≥ 21 18–20 ≤ 17	≥ 26 23–25 ≤22	≥ 23 20–22 ≤19	≥ 15 13–14 ≤ 12	≥ 25 21–24 ≤ 20	≥ 26 22–25 ≤21
Day 1	Control	14	8	16	15	11	11	20	11
	Smooth	15	9	16	15	11	11	20	11
	Rough	16	9	22	18	15	11	21	11
	SCV (Smooth)	20	10	21	20	15	11	22	12
Day 2	Smooth	15	9	16	15	11	11	21	11
	Rough	16	9	23	18	15	11	26	11
	SCV (Smooth)	20	9	22	20	15	11	22	12
Day 3	Smooth	15	9	16	15	11	11	21	11
	SCV (Smooth)	20	10	23	20	15	11	23	12
Day 4	SCV (Smooth)	22	10	22	20	18	11	23	12
	SCV (Rough)	22	10	23	27	21	12	28	12
Day 5	SCV (Smooth)	23	10	23	23	20	11	23	12
	SCV (Rough)	24	10	24	27	23	11	28	12

### Biofilm-forming ability

**Fig. 7:**
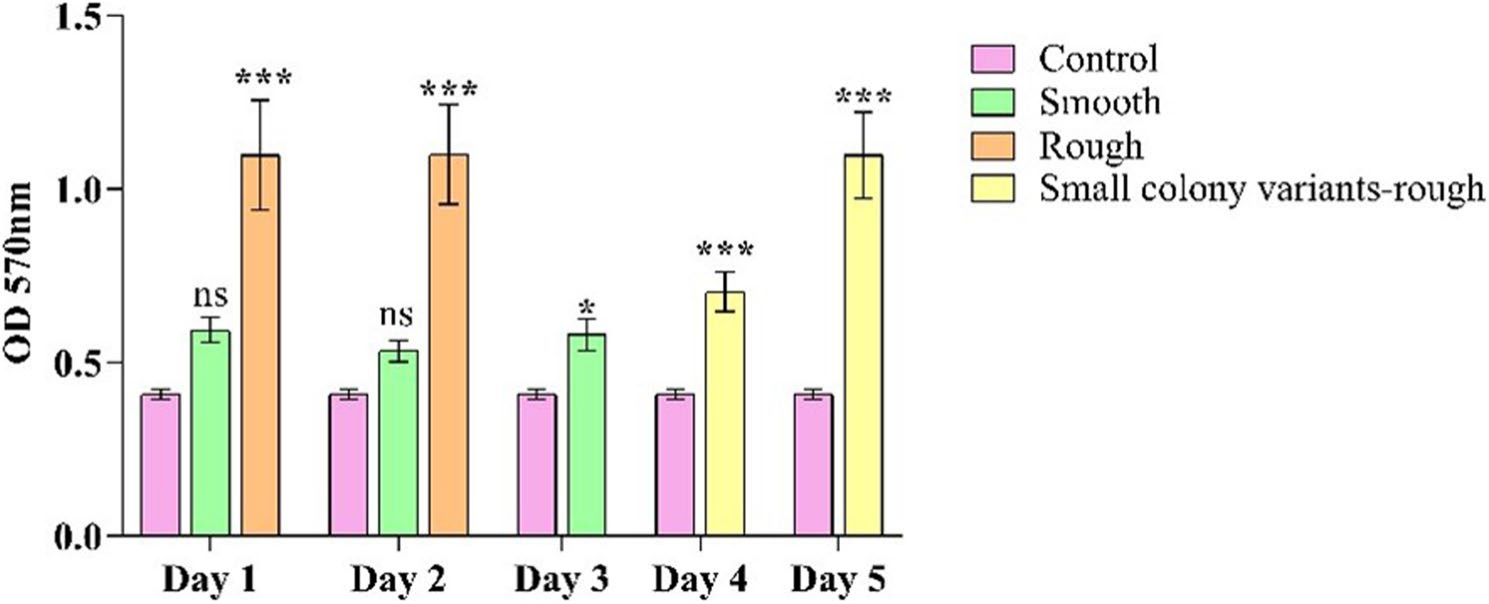
Assessment of biofilm-forming ability in colony morphotype variants. Biofilm formation was assessed after 24 h using the crystal violet (CV) assay. The stained biofilms were quantified by measuring absorbance at OD 570 nm using a microtiter plate reader. All experiments were performed in triplicate, and the data shown are represented as mean ± SD. *p* ≤ 0.05 compared to the control. (ns- not significant, **p ≤ 0.01, ***p ≤ 0.001)

The biofilm-forming ability of the variants was examined using a crystal violet assay. The wild type is a pre-identified biofilm-forming strain; the smooth morphotype (S) variant showed a slight increase in the OD value compared to the control, but remains lower than the other variants. Rough colony (R) morphotypes from days 1 and 2 exhibited strong biofilm formation. Small colony variants of smooth type (SCV-S) did not form biofilms, whereas the day 4 and 5 rough small colony variants (SCV-R) showed a significant increase in biofilm formation (Fig. 7).

## Discussion

In this study, the isolation of the lytic phage Disc against the Kleb_53 clinical strain revealed distinct phenotypic adaptations during phage resistance. The Disc phage demonstrated broad lytic activity and high stability, producing plaques with halo zones indicative of depolymerase activity. The phage had an icosahedral head and non-contractile tail, characteristic of the *Siphoviridae* morphology (outdated taxon). The adsorption time of the phage was 15 min with a latent period of 30 min and exhibited a notable burst size of 354 virions, larger than other* K. pneumoniae* phages like KP1801 (300 virions) and vB_KpnS_GH-K3 (291 virions) [31, 32]. It also showed a wide host range, lysing 48* Klebsiella* clinical strains, which is comparatively higher than other reported *Klebsiella* phages, UPM2146 phage lysing 5/22 [33], and vB_KpnP_Bp5 phage lysing 1/36 [34], further supporting its therapeutic potential.

During coevolution, distinct morphotypes such as rough and small colony variants emerged. The rough colony variant demonstrated larger plaque morphology and reduced halo zones, suggestive of capsular polysaccharide loss, a known receptor for phage adsorption. The emergence of heterogeneous colony types is consistent with earlier observations in phage-bacteria interactions [35, 36]. The dynamic shift in morphotypes likely reflects adaptive strategies, such as niche partitioning or survival trade-offs, possibly driven by differences in metabolic cost, spatial distribution, or environmental niches [37]. This phenotypic diversification may facilitate coexistence and long-term survival under phage pressure [38].

The coexistence of sensitive and resistant variants within the same culture suggests a complex adaptive balance. The unculturable colonies observed after day 5 may reflect a phage-pressure induced adaptation resulting in reduced metabolic activity or delayed growth, though not complete eradication, showing a dormant state [39]. This phenomenon is described as a viable but non-culturable (VBNC) state, as reported by Li et al. (2014) [40]. Phage resistance has also been associated with functional compensatory effects, such as decreased growth rates and impaired cellular functions. For example, Kortright et al. highlighted how resistance can lead to reduced cell division and function [41], which matches our findings of slower growth in the SCVs. These changes may be either stable mutations or reversible, transient adaptations based on environmental cues [42].

Further, loss of phage receptors such as capsule or LPS has been previously linked to reduced virulence and altered antibiotic interactions [43]. In our study, loss of capsule (evidenced by the absence of halo zones) appears to be a resistance mechanism, consistent with prior reports of LPS or capsule mutations contributing to phage resistance in *E. coli* and *Klebsiella* strains [43]. Similarly, research on bacteria coevolved with the lytic phage OMKO1 identified mutations in genes related to flagella, type IV pilus, and lipopolysaccharide biosynthesis—three of which were associated with phenotypic trade-offs in antibiotic resistance during coevolution [44, 45]. Resistance was also linked to altered biofilm phenotypes and changes in antibiotic sensitivity. Our phage-exposed variants showed variable biofilm formation, with some producing stronger biofilms, while others—particularly the smooth, small colony morphotypes—exhibited impaired biofilm development. This is consistent with findings in *E. faecalis* EFap02 phage-resistant mutants that displayed reduced biofilm production [46].

Interestingly, the emergence of phage-resistant morphotypes coincided with increased susceptibility to several antibiotics, including meropenem, ceftazidime, ceftriaxone, and cefotaxime/clavulanic acid. This “phage-induced antibiotic sensitivity” (PIAS) has been previously documented in *P. aeruginosa* strains, where phage resistance was accompanied by compromised efflux pump systems [47]. Also, the evolved phage-resistant strain R118-2 exhibited reduced virulence, diminished biofilm formation, and decreased aminoglycoside resistance compared to its wild-type counterpart, SMA118 [7]. In our case, reduced mucosity and possible loss of surface structures likely contributed to both phage resistance and resensitization to antibiotics [48]. This highlights the potential for phage therapy not only to target resistant bacteria but also to reverse certain antimicrobial resistance traits.

These results suggest that phage therapy can act as a dual-selective pressure, targeting bacteria directly while simultaneously reshaping bacterial phenotypes and susceptibility profiles. The observed morphological heterogeneity and associated growth-related trade-offs suggest that phage resistance often comes at a biological cost, potentially limiting the competitive advantage of resistant variants. Moreover, the dynamic and sometimes reversible nature of these changes highlights the complexity of bacterial adaptation under phage pressure. Phages may push bacteria to become less virulent and more sensitive to antibiotics, making the treatment more effective.

This study primarily focused on phenotypic observations. Although we documented clear changes in colony morphology, virulence, biofilm formation, and antibiotic sensitivity, the genetic and molecular underpinnings of these adaptations remain to be elucidated. Future work should include whole-genome sequencing, transcriptomic profiling, and single-cell analyses to dissect the mechanisms driving resistance and phenotypic heterogeneity. Additionally, prolonged incubation or serial passaging may help clarify whether observed fitness costs are due to temporary dormancy, persistent phage pressure, or stable genetic changes. Understanding these dynamics is critical for predicting the long-term outcomes of phage therapy and its integration into clinical settings.

## Conclusion

With renewed interest in phage therapy, understanding bacteria-phage interactions is essential. Phage resistance, an innate bacterial defense, poses a major challenge. This study highlights phenotypic changes in hypermucoviscous, hypervirulent *Klebsiella pneumoniae* during phage exposure, including shifts in colony morphology, loss of phage susceptibility and mucosity, antibiotic re-sensitization, reduced cell density, unculturability, and variable biofilm formation. These changes may unmask alternative receptors, aiding antibiotic effectiveness and infection clearance. The study underscores the importance of phenotypic insights in improving phage therapy, though the lack of genotypic correlation remains a key limitation.

## Data Availability

The sequences are submitted to the NCBI database (https://www.ncbi.nlm.nih.gov/), and the accession numbers are PV029729, PP994840, and PP994850.

## Abbreviations

CLSI: Clinical and laboratory standards institute
LB: Luria bertani
DAOL: Double agar overlay
PFU: Plaque-forming units
TEM: Transmission electron microscopy
MOI: Multiplicity of infection
OD: Optical Density
PSV: Phage-sensitive variant
PRV: Phage-resistant variants
EOP: Efficiency of plaquing
MRP: Meropenem
IPM: Imipenem
CEC: Cefotaxime/ clavulanic acid
CTR: Ceftriaxone
CAZ: Ceftazidime
PIT: Piperacillin-tazobactam
CIP: Ciprofloxacin
GEN: Gentamicin
CV: Crystal violet
SD: Standard deviation
MIC: Minimum inhibitory concentration
Hv: Hypervirulent
Hm: Hypermucoviscous
Kp: *Klebsiella pneumoniae*
R: Rough, dry, irregular colony
S: Smooth circular mucoid colony
SCV-S: Smooth small colony variants
SCV-R: Rough small colony variants
